# A Rare Case of Brachial Plexus Neuropraxia After COVID-19 Vaccination

**DOI:** 10.7759/cureus.21244

**Published:** 2022-01-14

**Authors:** Aditya Sharma, Anuj Gupta

**Affiliations:** 1 Orthopedics and Trauma, Max Super Speciality Hospital, Ghaziabad, IND; 2 Spine Surgery, Max Super Speciality Hospital, Ghaziabad, IND

**Keywords:** neuropraxia, steroids, vaccination, brachial plexus, covid

## Abstract

The brachial plexus injury is a rare complication after vaccination like that of the Influenza virus. Though a well-known and reported complication, there is still a dearth of literature mentioning its pathophysiology, the trend of involvement, symptoms, and treatment. This has also been reported after the coronavirus disease 2019 (COVID-19) vaccination. To the best of our knowledge, to date, only four cases have been reported so far. Every case needs to be reported to better understand the complication and formulate a line of management for better outcomes. We report a case of brachial plexus involvement after Covishield vaccination with complete recovery after treatment.

## Introduction

The brachial plexus is a network of nerves arising from the cervical spine and supply motor and sensory conduction to the upper limb. Though rare in adults, the most common cause of brachial plexus injury is trauma [1]. The incidence is about 4.2% of patients with motor vehicle collisions [1]. Rarely, the association of brachial plexus injury has been seen with vaccination, especially influenza vaccine [2]. One of the known viruses popular these days, i.e., coronavirus causing coronavirus disease 2019 (COVID-19) is no exception. We are presenting a case of brachial plexus neuropraxia and neuritis presenting as proximal muscle weakness of the arm and severe neuropathic pain.

## Case presentation

**Figure 1:**
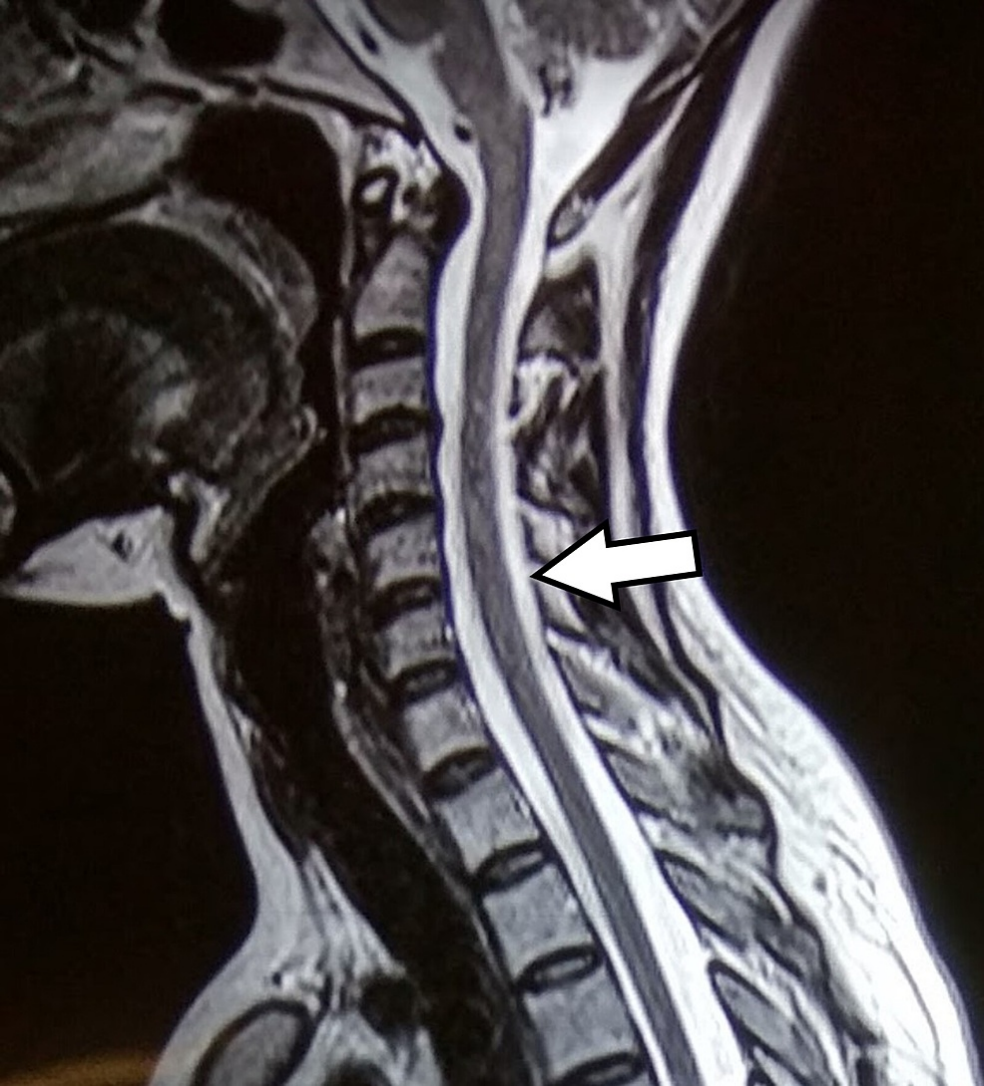
MRI (sagittal view) of the patient showing no obvious compression

A 34-year-old male with no known co-morbidities presented to the hospital with complaints of weakness in the left arm and pain in the left upper limb for one month. There was a history of COVID-19 vaccination (Covishield) immediately prior to the onset of symptoms. On examination, there was a loss of shoulder contour. There was the weakness of rotator cuff muscles especially supraspinatus muscle confirmed with positive empty can test and drop arm test. The patient was unable to abduct his arm even against gravity. The neurological examination of distal muscles to the elbow of the left upper limb was normal. The patient was further evaluated with x-rays of the cervical spine, magnetic resonance imaging (MRI) of the cervical spine, and electromyography (EMG) and nerve conduction velocity (NCV) study. The x-ray and MRI were clear with no obvious pathology (Figure 1).

The NCV showed involvement of the left axillary nerve. The EMG showed active denervation changes in the left triceps, biceps, deltoid, supraspinatus, rhomboids, and serratus anterior suggestive of the involvement of the left brachial plexus (Table 1). Since the application of COVID-19 vaccination precedes the onset of symptoms, the clinical diagnosis of post-vaccination brachial plexus neuropraxia was made. The patient was advised oral deflazacort 6mg thrice a day for 10 days, then twice a day for another 10 days, and then once a day for the last 10 days. On subsequent follow-ups, the neurology of the patient improved gradually and on the last follow-up after three months, there was a complete improvement as normal.

**Table 1:** Electromyography showing active denervation changes in left triceps, biceps, deltoid, supraspinatus, rhomboids, serratus anterior suggestive of the involvement of left brachial plexus MUAPs: motor unit action potentials

Muscle	Insertional	Spontaneous activity			Volitional MUAPs					Max volitional activity		
		Fibrillations	+wave	Fasciculations	Duration	Amplitude	Poly	Config	Recruitment	Amplitude	Pattern	Effort
1st dorsal interosseous left	Normal	None	None	None	Normal	Normal	None	Normal	Normal	Normal	Full	Max
Extensor indicis propius left	Normal	None	None	None	Normal	Normal	None	Normal	Normal	Normal	Full	Max
Flexor pollicis longus left	Normal	None	None	None	Normal	Normal	None	Normal	Normal	Normal	Full	Max
Pronator teres left	Normal	None	None	None	Normal	Normal	None	Normal	Normal	Normal	Full	Max
Biceps brachii left	Normal	None	None	None	Normal	Normal	None	Normal	Normal	Normal	Full	Max
Triceps brachii left	Normal	1+	None	None	Normal	Slightly increased	None	Normal	Incompact	Normal	Reduced	Sub max
Deltoid left	Normal	2+	None	Few	Normal	Normal	Few	Normal	Reduced	Normal	Reduced	Sub max
Supraspinatus left	Normal	None	None	None	Normal	Slightly increased	None	Normal	Reduced	Normal	Reduced	Sub max
Rhomboid major left	Normal	1+	None	None	Normal	Greatly increased	None	Normal	Normal	Normal	Full	Sub max
Serratus anterior left	Normal	None	None	None	Normal	Normal	None	Normal	Reduced	Normal	Reduced	Sub max
C5 paraspinal left	Normal	None	None	None	Normal	Normal	None	Normal	Normal	Normal	Full	Max
1st dorsal interosseous right	Normal	None	None	None	Normal	Normal	None	Normal	Normal	Normal	Full	Max

## Discussion

The brachial plexus neuropraxia is a known complication after influenza vaccination with neuropraxia occurring even after 28 days of injection. The cases have been reported as early as the same day of injection [3]. Even though it’s a known complication, there is a dearth of literature about its occurrence after the COVID-19 vaccination. Due to its rarity, the incidence is difficult to determine. For reference, only 18 cases have been reported so far between 2018 and 2020 after influenza vaccine administration. The total number of influenza vaccines administered during this period was 350 million [4]. The mechanism of its involvement in the brachial plexus is poorly understood.

To date, only a few case reports have been published about brachial plexus involvement after COVID-19 vaccination [5-8]. There is variation among these case reports regarding the onset of symptoms after vaccination. Queler et al. reported two cases, the symptoms started after 18 days in one, and in the other, it started only within 13 hours [8]. Other articles mention the onset of symptoms in four days [7], seven days [5], and nine days after injection [6]. In our patient, the symptoms started within a few hours of injection which increased in severity over three days. The patient developed weakness in proximal muscles of the left upper limb at which vaccine was given. There was weakness in shoulder abduction, flexion, and extension of the elbow. Fine movements of the hand and distal muscles were normal on examination. The patient also has severe pain on the lateral aspect of the shoulder and arm which couldn’t be explained with local pathology. The extent of involvement is different in different case reports. Mahajan et al. reported a case with the involvement of mainly distal muscles and Crespo Burillo et al. mentioned the involvement of all myotomes which include axillary, musculocutaneous, radial, and median nerve involvement [5,7]. In our patient, there is the involvement of only proximal muscles mainly supplied by nerve roots C5 and C6.

The diagnosis of brachial nerve involvement is mainly clinical but the involvement of the cervical spine has to be ruled out. As mentioned earlier, the exact mechanism of nerve involvement is not known, hence, no tests are available to establish the causality of COVID-19 vaccination and brachial plexus involvement. Most of the authors did nerve conduction studies to confirm the diagnosis made clinically and MRI cervical spine to rule out any degenerative pathology of the cervical spine. Also, a few authors got blood investigations too like C-reactive protein, erythrocyte sedimentation rate (ESR), antinuclear antibody, rheumatoid factor, Lyme antibodies, angiotensin-converting enzyme, herpes simplex antigen, and varicella-zoster virus antigen, etc. to rule out any inflammatory or other viral cause [5,7]. In our patient, we did electromyography and nerve conduction studies which confirmed the involvement of the brachial plexus. Also, an MRI of the cervical spine was normal in our case. One of the articles mentioned the use of MR neurography in such patients which further confirms the diagnosis [8].

The treatment of this condition is intravenous or oral steroids. Most of the authors have used oral steroids in involvement after the COVID-19 vaccination. Most of these patients have good results with recovery to near normal. The regimen can be different depending on the experience of the author. We used deflazacort on tapering dose for a month and there was complete recovery on follow-up. In our daily practice, we prefer deflazacort among steroids as it has shown higher efficacy and good tolerability compared to other steroids [9].

## Conclusions

Brachial plexus neuropraxia is a rare but known complication after influenza vaccination. With widespread vaccination of COVID-19, this complication is reported from different parts of the world, and knowledge of this may help healthcare workers in managing it. We recommend the use of EMG, NCV, and MRI of the cervical spine in a patient with clinical suspicion of brachial plexus involvement and a history of COVID-19 vaccination. Early use of steroids has better outcomes.
